# PIWI-interacting RNAs in cancer: Biogenesis, function, and clinical significance

**DOI:** 10.3389/fonc.2022.965684

**Published:** 2022-09-23

**Authors:** Jie Yao, Mei Xie, Xidong Ma, Jialin Song, Yuanyong Wang, Xinying Xue

**Affiliations:** ^1^ Department of Respiratory and Critical Care, Beijing Shijitan Hospital, Capital Medical University, Beijing, China; ^2^ Department of Respiratory and Critical Care, The Chinese People's Liberation Army of China (PLA) General Hospital, Beijing, China; ^3^ Department of Respiratory and Critical Care, Weifang Medical College, Weifang, China; ^4^ Department of Thoracic Surgery, Tangdu Hospital of Air Force Military Medical University, Xi’an, China

**Keywords:** piRNA, PIWI, cancer, biomarkers, diagnosis, prognosis

## Abstract

PIWI-interacting RNAs (piRNAs) are a less-studied class of small non-coding RNAs approximately 24–31 nucleotides in length. They express in germline and somatic cells and form complexes with PIWI proteins to exert regulatory effects. New studies show that piRNAs are aberrantly expressed in various cancers. In this review, we focus on those piRNAs that are associated with cancer hallmarks such as proliferation, invasion, and chemoresistance and discuss their potential as biomarkers for cancer diagnosis and prognosis.

## Introduction

The Human Genome Project shows that over 95% of the human genome is comprised of non-coding RNAs that do not code proteins, known as the “dark” or “junk” RNA of the genome (1). In recent years, this research area has entered the forefront due to the active role of non-coding RNAs in life activities and disease development. Non-coding RNAs are classified by length into long non-coding RNAs (lncRNA, >200 nt) and small non-coding RNAs (<200 nt) (2).When it comes to small non-coding RNAs, people usually think of miRNAs (3, 4), siRNAs (5), and other small RNAs that have been widely studied. In fact, piRNAs, a class of non-coding RNAs, have also been a hot topic in molecular research (6).

piRNAs were first identified in *Drosophila* testis 20 years ago and derived from the Su (Ste) locus (7, 8). Compared with miRNAs and siRNAs, piRNAs are longer (24–31 nt in length) and more abundant (approximately 5 × 10^4^ species) (9, 10). Its production is not dependent on RNase-like nucleases and has a 3′ terminal 2′O-methylation modification (6, 9). In addition, miRNAs and siRNAs are processed from double-stranded RNA (dsRNA) precursors *via* Dicer, whereas piRNAs are derived from single-stranded RNA (ssRNA) precursors of piRNA clusters (11). piRNAs, along with miRNAs and siRNAs, are all the critical cores of RNA interference (RNAi), forming RNA-induced silencing complexes (RISC) with Argonaute family proteins that recognize and regulate target expression through full or partial Watson–Crick base pairing (12, 13). Argonaute proteins are divided into two subfamilies: the AGO proteins and the PIWI proteins—the latter being another spotlight in this review (14). Numerous studies confirm that PIWI family proteins are essential for piRNA biogenesis. Like piRNAs, PIWI proteins were first discovered in *Drosophila melanogaster* and regarded as key genes for germline development. In *Drosophila*, PIWI proteins are classified as PIWI, Aub, and Ago3, all of which play key roles in piRNA biogenesis. PIWI regulates transposon transcription mainly in the nucleus. In contrast, Aub and Ago3 act in the cytoplasm to cleave transposon transcripts (15). In mice, PIWI proteins are divided into three classes, PIWIL1 (MIWI), PIWIL2 (MILI), and PIWIL4 (MIWI2), all of which are expressed at different stages of spermatogenesis (16, 17). In humans, PIWI proteins are divided into four isoforms—PIWIL1 (HIWI1), PIWIL2 (HILI), PIWIL3 (HIWI3), and PIWIL4 (HIWI2)—with highly conserved properties (18, 19).

It was previously thought that piRNAs were mainly found in gonadal cells (20). However, recent studies have shown that many piRNAs are also expressed in somatic cells, and abnormal piRNA expression has been found in many diseases (21–23). The dysregulation of piRNAs is also present in various human cancers, suggesting that these piRNAs may be involved in cancer progression (24, 25). The biological functions of piRNAs in germline are well understood, but their roles in cancer cells remain unclear. Based on available findings, this review briefly summarizes the biogenesis of piRNAs. This is followed by a series of studies on piRNAs with various cancer hallmarks and a discussion of their prospects as diagnostic and prognostic markers for cancer.

## Biogenesis of piRNA

**Figure 1:**
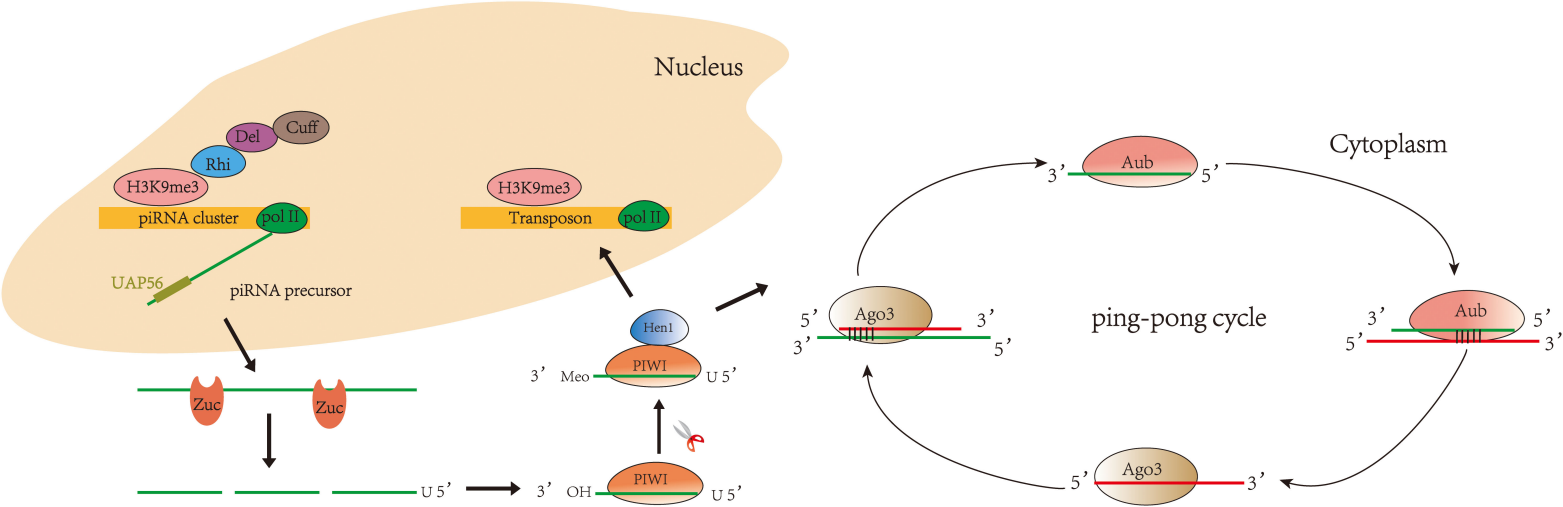
Biogenesis of piRNA: primary pathway and secondary pathway. In the primary pathway, piRNA forms a complex with PIWI proteins to mediate transposon silencing. In the secondary pathway, piRNA binds to Aub to produce secondary piRNA, while secondary piRNA binds to Ago3 to produce primary piRNA, and so the cycle continues.

Currently, studies on the biogenesis of piRNAs are mainly focused on organisms such as *Drosophila*, mice, silkworm, and *Caenorhabditis elegans* (26–31). The biogenesis of piRNA varies slightly in different organisms, but the main processes are conserved. piRNAs fall into three main categories based on their source: piRNAs from transposon, piRNAs from mRNA 3′UTR, and piRNAs from long non-coding RNAs (32). Two major pathways are currently recognized in piRNA biogenesis (**Figure 1**): primary and secondary (“ping-pong cycle”). The primary pathway occurs in somatic and germ cells. The secondary pathway (“ping-pong cycle”) occurs only in germ cells (33, 34). piRNA clusters are marked by histone H3 lysine 9 (H3K9me3) and transcribed by RNA polymerase II (RNA Pol II) (35). Rhino (Rhi), a member of the HP1 protein family, then specifically binds to the H3K9me3 marks on the piRNA clusters and recruits Cutoff (Cuff) through the adaptor protein Deadlock (Del) (36, 37). A protein complex comprising Rhi, Del, and Cuff ensures the continuous extension of piRNA cluster transcription (34). piRNA clusters are transcribed into piRNA precursors in the nucleus (38). Then, piRNA precursors are transported to the cytoplasm *via* UAP56 and cleaved by an endonuclease Zucchini (Zuc) (39–41) to produce short-stranded piRNA intermediates with 5′ uracil. After binding to PIWI proteins, piRNAs are further trimmed by 3′–5′ exonuclease Trimmer (42–46) and methylated by Hen1 enzyme to produce mature piRNA/PIWI complexes (47–49). The PIWI/piRNA complexes return to the nucleus and interact with transposon transcripts through base pairing. PIWI/piRNA inhibit transposon expression there through H3K9me3. Notably, piRNAs amplify *via* the secondary pathway in *Drosophila*, zebrafish, and most mammalian germline (50, 51)—for example, in *Drosophila* germ cells, piRNAs bind to PIWI subfamily Aub protein to form complexes. Then, piRNA/Aub complexes recognize and target transposon sequences and generate secondary piRNAs. When the secondary piRNAs are loaded to Ago3 (52, 53), the transposon transcripts will be cleaved and produce new piRNAs identical to the primary piRNAs. This pathway is also known as the ping-pong cycle.

## piRNAs and cancer

**Figure 2:**
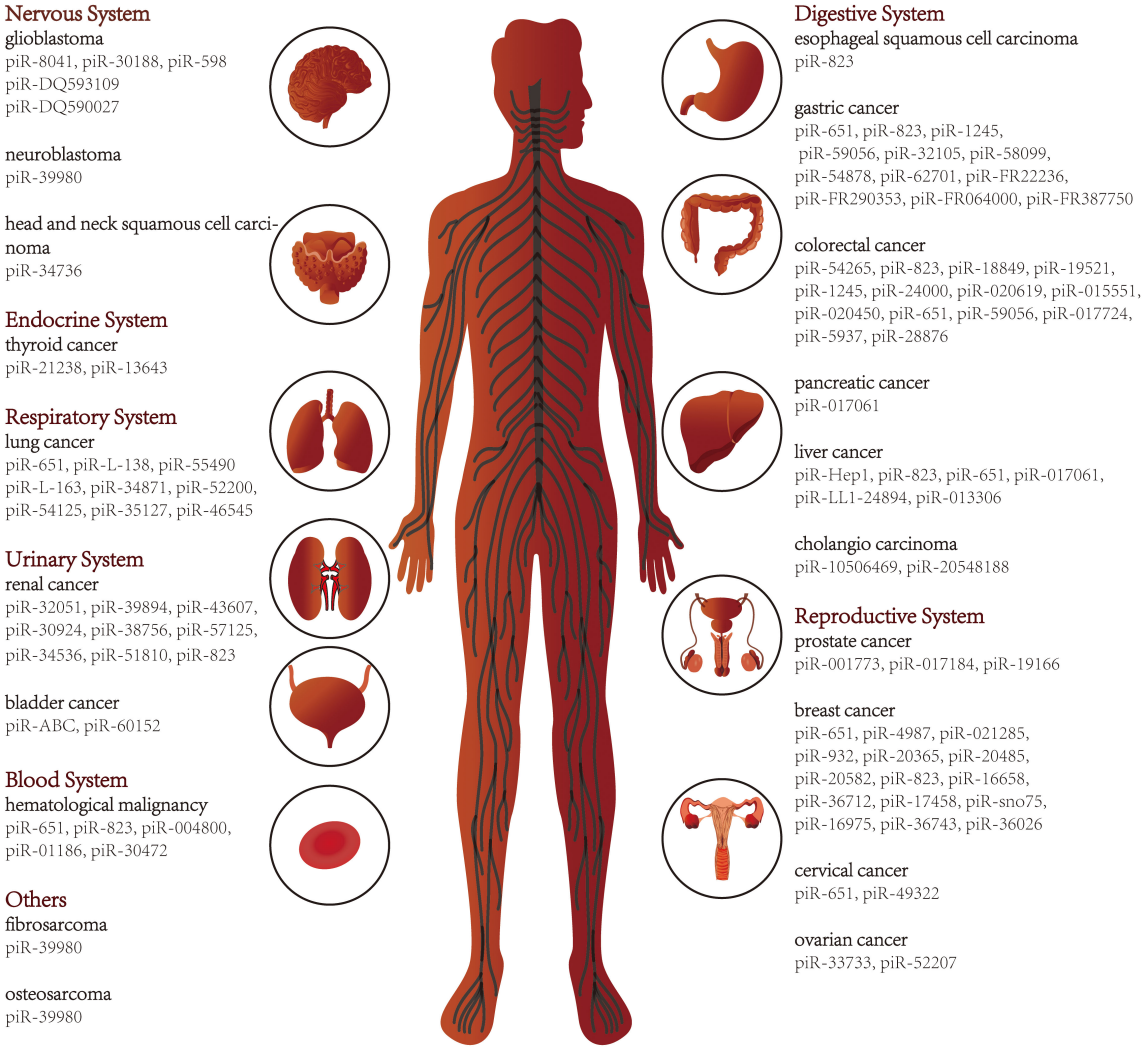
piRNAs in various types of cancer.

With the development of sequencing technology, piRNAs in various diseases, including cancer, have been progressively deciphered. Here we review studies of piRNAs in different systems of human cancers (**Figure 2**) and further explore the relationship between piRNAs and various hallmarks of cancer. In addition, piRNAs have been suggested as cancer diagnostic tools, therapeutic targets, and prognostic biomarkers. As piRNA binding proteins, the PIWI proteins also receive attention in this review.

### piRNAs and proliferation, migration/invasion

Uncontrolled cell proliferation is a major hallmark of cancer, and piRNAs are involved (54). Deep sequencing analysis confirmed that piR-651 was upregulated in lung cancer tissues and cell lines and promoted cell proliferation and tumor formation through the upregulation of cyclinD1 and CDK4 expression (55, 56), although the exact mechanism is not clear. In addition, the overexpression of piR-8041 in glioblastoma multiforme reduced cell proliferation, and pretreatment with piR-8041 *in vivo* significantly reduced the volume of intracranial mouse xenograft tumors (57). Cheng et al. pointed out that piR-823 was downregulated in gastric cancer tissues and cells, and its mimics increased the level of piR-823 and significantly inhibited tumor growth in a dose-dependent manner (58).

Tumor cell proliferation is usually a promoter of cell metastasis. Cancer cells leave the primary site and colonize outside the primary tumor, while metastatic cells also proliferate, migrate, and invade surrounding tissues (59, 60). This cascade is the main cause of poor prognosis, and piRNAs are also involved in some stages of this process. In metastatic and non-metastatic clear cell renal cell cancer (ccRCC), piR-30924 and piR-38756 were more highly expressed in metastatic tumors, while piR-57125 was less expressed in metastatic tumors. More importantly, their differential expression was associated with tumor recurrence and overall survival (OS) and considered to be an independent prognostic factor (61). As piRNA-binding proteins, the PIWI subfamily also functions in the metastatic properties of cancer. Reports have shown that PIWIL1 downregulation significantly reduced the proliferation, migration, and invasiveness of hepatocellular carcinoma (HCC) (62). What is more, PIWIL1 was much more abundant in endometrial cancer tissues compared with atypical hyperplasia and normal tissues. The overexpression of PIWIL1 exerted an influential role in maintaining stem cell-like characteristics, including enhanced tumor cell viability, migration, invasion, and sphere formation activity. Furthermore, PIWIL1 was related to increased mesenchymal markers and E-cadherin inhibition (63). Tumor metastasis involves many steps, but each step is not independent. Epithelial–mesenchymal transition (EMT) is an essential step in tumor development (64). After the tumor cells underwent EMT, they acquired invasion characteristics and infiltrated into the surrounding stroma, forming a microenvironment that promoted tumor growth and metastasis (65–67). The discovery of PIWI proteins correlated with EMT markers may have profound implications for improving treatment methods and controlling tumor metastasis. piRNAs may also have some important role in the EMT process.

### piRNAs and cell cycle

A large number of regulatory factors are involved in the cell cycle process of tumor cells. If a regulatory factor is abnormal, the cell will not be able to move from one stage to the next, which is called cell cycle arrest (68). Cell cycle arrest is a prominent feature of tumor progression. Many piRNAs and PIWI proteins are closely related to cell cycle arrest. Antisense sequence inhibited piR-823 in colorectal cancer (CRC) cells HCT116 and DLD-1, followed by flow cytometry showing the increased G1 phase cell population (69). Liu et al. found that PIWIL1 was overexpressed in gastric tissues and cell lines, and the downregulation of endogenous PIWIL1 by antisense or RNAi effectively inhibited the proliferation and G2/M phase cell cycle arrest in gastric cancer cells (70). In addition, cyclinD1, an indispensable driver of cell cycle progression, controlled the secretion of piRNAs (piR-016658 and piR-016975) in breast cancer (71).

### piRNAs and genetic variation

Although most studies have focused on the aberrant expression and function exploration of piRNAs, some studies have still noted the association between piRNAs and genetic variants (72). Single-nucleotide polymorphisms (SNPs) are the most common genetic variations (73). Chu et al. found that reference SNP rs11776042 (T > C) in piR-015551 participated in the development of CRC (74). Furthermore, SNPs in PIWI are more common and more strongly associated with cancer risk. A case–control study by Sung et al. found the pronounced protective effect of PIWIL1 rs11060845 in progesterone receptor-positive breast cancer patients. At the same time, two SNPs in PIWIL1 (rs4759659 and rs11060845) were correlated with disease-free survival (DFS). Another study found that an SNP in PIWIL1, rs10773771 (CT/CC), reduced the risk of HBV-associated HCC (75). Tumor risk assessment can be achieved by identifying SNPs in piRNAs and PIWI proteins.

### piRNAs and epigenetic modification

The main modes of epigenetic modification include DNA methylation, histone modifications, and chromatin remodeling (74–78). DNA methylation is closely related to oncological diseases, and CpG island methylation can lead to the transcriptional inactivation of oncogenes. Recent studies report that piRNAs are involved in CpG island methylation (76). In breast cancer, piR-021285 suppressed ARHGAP11A expression by facilitating methylation at a CpG site within the 5′UTR/first exon. ARHGAP11A is a known pro-apoptotic regulator, and its reduced expression leads to the inhibition of apoptosis (77). In breast cancer stem cells, piR-932 and PIWIL2 formed a complex to promote CpG island methylation of the Latexin promoter region and reduced its expression. Latexin is a tumor suppressor that reduces the transformation of senescent stem cells into cancer stem cells, showing a cancer-suppressive ability (78). A study demonstrated that PIWIL2 played an essential role in the transformation of cervical epithelial cells to tumor-initiating cells *via* epigenetics-based cell reprogramming (79). In addition, PIWIL1 mediated PTEN hypermethylation *via* DNA methyltransferase 1 in type I endometrial cancer (80). DNA methylation is mainly mediated by DNA methyltransferases, which consist of two major classes, DNMT3 (DNMT3a and DNMT3b) and DNMT1, that catalyze and maintain DNA methylation, respectively (81, 82). Except for DNA methylation, piRNAs and PIWI proteins are also closely related to histone modifications. piRNAs have the ability to promote histone modifications in *D. melanogaster* (83). In addition, PIWI proteins have been shown to directly influence histone modifications at piRNA targets (84, 85). As an important class of histone modifications, ubiquitination plays a very important role in protein localization, metabolism, regulation, and degradation. The association of ubiquitination with non-coding RNAs, including piRNAs, has also received much attention (86). In CRC, piR-823 repressed the ubiquitination of hypoxia-inducible factor 1α by upholding the expression of glucose 6 phosphate dehydrogenase, ultimately upregulating glucose consumption and suppressing intracellular reactive oxygen species content (87). The role of piRNAs in cancer often involves more than one cancer hallmark—for example, in multiple myeloma (MM), piR-823 inhibited tumor formation *in vitro* and *in vivo* and induced the expression of cell cycle regulators and apoptosis-related proteins as well as the secretion of vascular endothelial growth factor. More notably, piR-823 inhibition was also linked to the expression downregulation of *de novo* DNA methyltransferases DNMT3A and DNMT3B, the latter leading to the re-expression of the methylation-silenced tumor suppressor gene p16 (88). These diverse functions may harbor complex mechanisms that deserve further exploration.

### piRNAs and chemotherapy

Chemotherapy is the most widely used treatment for malignant tumors (89). Some piRNAs are associated with chemotherapeutic efficacy and even have synergistic anticancer effects, providing new insights into how we can use piRNAs well to enhance chemoresistance or sensitivity. Recently, a study identified that piR-L-138 was a key factor of cisplatin resistance in patients suffering from lung squamous cell carcinoma (90). In addition, Tan et al. found that the upregulation of piR-36712 in breast cancer displayed a synergistic anticancer effect with chemotherapy drugs (24). Another interesting finding was that CRC cells with piR-54265 overexpression were resistant, while piR-54265 knockdown was sensitive to 5-FU and oxaliplatin, two first-line drugs commonly used in chemotherapy for advanced CRC (91). This finding will be significant for improving the efficacy of chemotherapy. Another study conducted by Roy et al. observed that the overexpression of piR-39980 decreased the sensitivity of the anticancer drug doxorubicin and inhibited the doxorubicin-mediated apoptosis of neuroblastoma cells (92). The discovery that piRNAs could regulate chemosensitivity sparks new hope for cancer therapy. However, little is known about its underlying regulatory mechanisms, and further research is needed in the future.

### piRNAs in liquid biopsy

Liquid biopsy is considered an ideal channel for developing cancer biomarkers (93, 94). Common liquid specimens include blood, urine, cerebrospinal fluid, thoracoabdominal fluid, and bronchoalveolar lavage fluid. Compared with invasive examination, these specimens have the advantages of convenient collection, reliable repeatability, and high patient acceptance. Many studies have found abnormal piRNAs in the body fluids of cancer patients—for example, piR-823 was downregulated in the tissue but upregulated in the serum and urine of patients with renal cell carcinoma (RCC) (95). The piR-65 expression in different samples was also inconsistent. Cheng et al. suggested that piR-651 was upregulated in gastric cancer tissues, affecting cell growth and cell cycle arrest (96). On the contrary, Cui et al. showed that piR-651 was downregulated in the peripheral blood of gastric cancer patients with diagnostic significance (97). Interestingly, piR-651 expression was downregulated in the serum samples from classical Hodgkin’s lymphoma patients at diagnosis but rose to levels similar to healthy patients after complete remission, suggesting that piR-651 could be an indicator of long-term monitoring and disease progression (98). The same molecule was differentially expressed in different samples (tissue and blood) and before and after treatment. These findings prompted researchers to consider the possibility that piR-651 detected in the serum was derived from circulating cells rather than tumor cells. Zhou et al. also reported that a high piR-1245 expression was observed in the gastric juice of gastric cancer patients with poor OS, and the area under the curve (AUC) was 0.885, indicating that piR-1245 in gastric juice was both a diagnostic and prognostic indicator (99). Some other findings suggested that piR-823 mainly accumulated in the extracellular vesicles (EVs) of peripheral blood from MM patients and EVs from MM cells (MM-derived EVs). Among them, MM-derived EVs were able to effectively transfer piR-823 to EA.hy926 endothelial cells and alter their biological characteristics, such as cell proliferation, tube formation, and invasion (100). EVs are an intercellular communication tool that plays an essential role in information transmission and tumor microenvironment (101, 102). piRNAs encapsulated in EVs can be stably present in body fluids and have the potential to be promising markers for cancer diagnosis and prognosis.

### piRNAs and immunity

Research on piRNAs has also been carried out in other areas such as immune regulation. It has been found that piR-30840 complementarily bound to pre-mRNA intron to inhibit IL-4 expression and Th2 T-lymphocyte development (103). In another study, myeloid-derived suppressor cells increased the production of piR-823, thereby enhancing the stemness of MM stem cells (104). Research on immune cell regulation based on piRNAs is still in its infancy, and it is worthwhile to explore more piRNAs in the tumor microenvironment.

## Clinical significance of piRNAs in cancer

### piRNAs as diagnostic biomarkers in cancer

For any malignancy, it would be of paramount significance to achieve early diagnosis. It is no longer sufficient to detect only traditional markers for the early diagnosis of various tumors. In some studies, piRNAs show a better diagnostic ability than traditional markers—for instance, piR-13643 (AUC = 0.821) and piR-21238 (AUC = 0.823) performed better than the currently used biomarker HBME1 in distinguishing malignant nodules from the benign ones and may serve as new biomarkers for an accurate detection of papillary thyroid carcinoma (105). In addition, serum piR-5937 and piR-28876 were able to distinguish CRC from healthy controls with very high sensitivity and specificity. The detailed values are shown in **Table 1**. Their diagnostic characteristics were compared with the traditional markers CEA and CA199. In comparison, alterations in piR-5937 and piR-28876 were detected in 71 and 69% of colon cancer patients, respectively, while CEA and CA199 alterations were detected in only 48 and 26% of patients, respectively. The highest diagnostic sensitivity (86%) was achieved with the combination of CEA, CA199, and both piRNAs (107). Accumulating studies have shown that piRNAs are good diagnostic markers in cancer (**Table 1**). Although a single piRNA is sufficient to distinguish cancer patients from healthy controls, combined biomarkers may be more diagnostically accurate than an individual biomarker. Qu et al. found that five differentially expressed piRNAs (piR-001311, piR-004153, piR-0177n 23, piR-017724, and piR-020365) performed better than CEA in CRC detection, with an area under the curve of 0.876 (108).

**Table 1:** piRNAs as diagnostic or prognostic biomarkers in clinical studies.

Cancer type	Sample number	Source	piRNA	Expression	AUC	Sensitivity	Specificity	OS (*p*-value)	PFS (*p*-value)	Reference
Gastric cancer	66 patients, 66 controls	Gastric juice	piR-1245	Up	0.885	0.909	0.742	0.015	0.013	(106)
Gastric cancer	93 patients, 32 controls	Peripheral blood	piR-651	Down	0.841	0.709	0.813	/	/	(97)
			piR-823	Down	0.822	0.805	0.812	/	/	
Colorectal cancer	80 patients, 80 controls	Serum	piR-5937	Down	0.806	0.718	0.725	/	/	(107)
			piR-28876	Down	0.807	0.753	0.700	/	/	
Colorectal cancer	120 patients, 120 controls	Serum	piR-017724	Down	0.756	0.575	0.817	0.005	0.002	(108)
Colorectal cancer	725 patients, 1,512 controls	Serum	piR-54265	Up	0.896	0.857	0.651	/	/	(109)
Colorectal cancer	140 patients, 140 controls	Serum	piR-020619	Up	0.871	0.843	0.764	/	/	(110)
			piR-020450	Up	0.841	0.814	0.750	/	/	
			piR-020814	Up	0.680	0.586	0.729	/	/	
Colorectal cancer	87 patients, 87 controls	Tissue	piR-24000	Up	0.818	0.931	0.690	/	/	(111)
Esophageal squamous cell carcinoma	54 patients, 54 controls	Tissue	piR-823	Up	0.713	0.630	0.770	/	/	(112)
Clear cell renal cell carcinoma	118 patients, 75 controls	Tissue	piR-34536	Down	0.815	0.780	0.781	0.005	0.040	(113)
			piR-51810	Down	0.829	0.856	0.712	<0.001	0.001	

AUC, area under the curve; OS, overall survival; PFS, progression-free survival.

### piRNAs as prognostic biomarkers in cancer

Like other non-coding RNAs, piRNAs were associated with the prognosis of cancer patients. A total of 132 piRNAs were detected in the transcriptional atlas of the gastric cancer genome, and nearly half of them were overexpressed. Among them, piR-FR222326 was associated with OS and five piRNAs (piR-FR157678, piR-FR387750, piR-FR381169, piR-FR290353, and piR-FR064000) were significantly associated with recurrence-free survival (RFS) (114). Data from Zhao et al. suggested that HIWIL1 may be a useful prognostic factor for patients with HCC after curative resection, especially in the case of low serum AFP and low pathological grading (115). Lliev et al. also observed no difference in PIWIL2, PIWIL3, and PIWIL4 expression between RCC and renal parenchyma. However, their expression gradually decreased together with increasing clinical stage (116). Although the functional mechanisms of these piRNAs remain unclear, their expression is correlated with patient survival, tumor stage, or other clinical parameters. This suggests their potential as tumor prognostic markers.

## Conclusion

Since the discovery of piRNAs in 2001, their critical role in different species and different conditions of physiology and pathology has been extensively reported. This paper reviews the advances in piRNA studies and provides a complete description of piRNA biogenesis, function, and clinical implications in cancer. Under normal conditions, piRNAs are synthesized and degraded in germ cells and somatic cells at relatively stable levels. However, when the piRNA expression is disrupted, they lose normal function and may lead to cancer development. Some trials have confirmed that piRNAs are aberrantly expressed within various cancers in a cancer-specific manner. Some piRNAs are also found in the blood or urine of cancer patients as diagnostic or prognostic indicators. Most reports are limited to their effect on tumor phenotype, and only a tiny fraction have investigated their mechanisms, leaving many unanswered questions. As candidate biomarkers, piRNAs require multi-center cohort studies with large samples to facilitate their clinical application in human cancer. Furthermore, although piRNAs are considered therapeutic targets, their association with available clinical therapies is mainly focused on chemotherapy. The role of piRNAs in radiotherapy and immunotherapy has not been explored. The comprehension of piRNAs is still in its infancy, and the development of multi-omics and high-throughput sequencing technologies is expected to help us gain a more comprehensive understanding. It is hoped that the description of this review will inspire more research and clinical trials to unlock the potential biological code of piRNAs.

## Author contributions

JY searched for literature and drafted this manuscript. MX reviewed the manuscript and polished the grammar. XM, JS and YW performed extensive literature search and discussion. XX designed and revised the manuscript. All authors contributed to the article and approved the submitted version.

## Funding

This work was supported by the National Natural Science Foundation of China (62176166, 62076254).

## Conflict of interest

The authors declare that the research was conducted in the absence of any commercial or financial relationships that could be construed as a potential conflict of interest.

## Publisher’s note

All claims expressed in this article are solely those of the authors and do not necessarily represent those of their affiliated organizations, or those of the publisher, the editors and the reviewers. Any product that may be evaluated in this article, or claim that may be made by its manufacturer, is not guaranteed or endorsed by the publisher.

## Abbreviations

piRNAs, PIWI-interacting RNAs; dsRNAs, double-stranded RNAs; ssRNAs, single-stranded RNAs; RNAi, RNA interference; RISC, RNA-induced silencing complex; ccRCC, clear cell renal cell cancer; RCC, renal cell carcinoma; HCC, hepatocellular carcinoma; EMT, epithelial–mesenchymal transition; SNPs, single-nucleotide polymorphisms; CRC, colorectal cancer; DFS, disease-free survival; AUC, area under the curve; EVs, extracellular vesicles; MM, multiple myeloma; OS, overall survival; RFS, recurrence-free survival; H3K9me3, histone H3 lysine 9; RNA Pol II, RNA polymerase II.
